# Sir William Banks on the Origin of Cancer

**Published:** 1900-03-17

**Authors:** 


### SIR WILLIAM BANKS ON THE ORIGIN OF CANCER.

In delivering the first of the Lettsomian Lectures before the Medical Society Sir William Banks addressed himself principally to the question of cancer of the breast, but he dealt with the matter in such a manner that many of his remarks apply almost equally to cancer of other parts, and this is especially true of what he said in regard to the relation of the disease to diet. Certain it is, he says, that the disease has not appeared to affect the weakling, the tea-shrivelled, mummy-like old maid, or the ill-fed, more than half-starved drunkard who never has any money to spend on good food. Its most numerous victims are well-nourished persons, with plenty of beef and fat about them, and often with a fine healthy colour in their cheeks. The better the nutrition, and the younger the patient, the more deadly and rapidly growing is the cancer. Take what is termed a fine "buxom" woman of 30 or 35 with cancer of the breast, and her chance of permanent cure, even by the most extensive of operations, is almost nil. Being satisfied, then, that well-nourished, well-developed, healthy people are the most numerous victims of cancer, the question at once arises, Is the increase of cancer in any way synchronous with an increase of nutrient material—that is, of food—throughout the country? There cannot, he says, be a doubt of it. Ever since the passing of the corn laws bread has been cheap and plentiful, while during the last 20 years the importation of animal food from other countries has been enormous. Our working classes fare admirably, while our better classes eat infinitely too much, especially of animal food. "I am pretty well convinced," he says, "that when a man is over 45 excess in food is perhaps worse for him than excess in drink," and he holds that while the result of taking too much nourishing food is the production of a widely spread second-rate kind of gout of a very different type from the acute kind produced in former days by the copious drinking of beer and port wine, so another effect of the same over-eating is the production of a cancerous diathesis; and he points out that, although the researches made by Mr. Haviland in regard to the topographical distribution of the disease do undoubtedly show the existence of a tendency for cancer to predominate in certain areas, it may be that such areas are also those in which people are best nourished. Of course there is a stock answer to all this, namely, that vegetarians and rice eaters are just as subject to cancer as gross feeders and flesh eaters. This, however, Sir William Banks will not admit. Cancer, he says, is not so common in Ireland, where feeding has been poor and very largely of a vegetable character, and where the improvement of living has not proceeded *pari passu* with that in England. From what can be learnt of the whole continent of Africa that vast country enjoys a remarkable immunity from the disease, especially the Egyptian area and the northern parts. At the Cape, also, cancer although common among the whites is very rare among the natives, and he gives many other examples tending in the same direction. After going through the various causes to which the increase of cancer has been attributed by various observers, and negativing them one by one, he says, "What great change, then, has taken place in the national life? Richer and more abundant food. You will note particularly that it is the male who eats the heavy food in ever increasing quantities, while the female remains much as before in her eating, and note along with this that it is the men now who suffer most severely from cancer, and not women as formerly. Again, in all England the most tainted places are London, the Thames Valley, and the counties adjoining the metropolis where the most luxurious habits and the best living prevail, and it has been pointed out that even in the capital itself, the parts where those in easy, comfortable circumstances, who are supplied with more than the necessaries of life, abide, show the highest mortality." He further points out that amongst the very highest mortality rates for occupations are those which include commercial travellers, coachmen and grooms, merchant seamen, maltsters, brewers, inn-keepers, butchers, and plumbers, persons who are likely to eat and drink abundantly and do not work off their spare products. As to the immediate or exciting cause that is another matter. The point raised is that, influencing and deciding the effectiveness of such causes, there is a diathesis or condition of body in which the tendency to the development of cancer is more strongly marked than in other conditions, and that this particular bodily state is connected with an excessive consumption of food. The process may be microbic, the localising influence may be traumatism or other cause of lessened resistance; who knows?

